# Sex-specific associations between dietary legume subtypes and type 2 diabetes in a prospective cohort study

**DOI:** 10.4178/epih.e2024083

**Published:** 2024-10-17

**Authors:** Hye Won Woo, Sangmo Hong, Min-Ho Shin, Sang Baek Koh, Hyeon Chang Kim, Yu-Mi Kim, Mi Kyung Kim

**Affiliations:** 1 Department of Preventive Medicine, Hanyang University College of Medicine, Seoul, Korea; 2 Institute for Health and Society, Hanyang University, Seoul, Korea; 3 Division of Endocrinology and Metabolism, Department of Internal Medicine, Hanyang University Guri Hospital, Hanyang University College of Medicine, Guri, Korea; 4 Department of Preventive Medicine, Chonnam National University, Medical School, Gwangju, Korea; 5 Department of Preventive Medicine and Institute of Occupational Medicine, Yonsei Wonju College of Medicine, Wonju, Korea; 6 Department of Preventive Medicine, Yonsei University College of Medicine, Seoul, Korea

**Keywords:** Legumes, Soybeans, Sex factors, Diabetes mellitus type 2, Prospective studies, Republic of Korea

## Abstract

**OBJECTIVES:**

Dietary soy, known for its high phytoestrogen content, has been suggested to exhibit a sex-specific association with type 2 diabetes. However, evidence regarding the sex-specific associations of different legume subtypes with type 2 diabetes remains scarce. We aimed to evaluate whether habitual consumption of soy and non-soy legumes (beans and peanuts) was prospectively and sex-specifically associated with the risk of type 2 diabetes incidence, taking into considering significant sex-specific genetic factors beyond legume consumption.

**METHODS:**

A total of 16,666 participants (96,945 person-years) were followed and 945 incident cases were observed. Cumulative intake of legume subtypes was calculated using a food frequency questionnaire administered at baseline and during the revisit surveys.

**RESULTS:**

Non-soy legumes are inversely associated with type 2 diabetes in both men and women. Dietary soy intake, however, demonstrated a unilaterally interacting sex-specific association with type 2 diabetes risk (p_interaction_ for sex=0.017). Specifically, there was a significant inverse association with type 2 diabetes risk in women (incidence rate ratio, 0.66; 95% confidence interval, 0.48 to 0.80; p_trend_=0.007), but no such association was observed in men. This sex-specific association persisted and even appeared antagonistic in minor allele carriers of 2 novel single nucleotide polymorphisms, rs10196939 (*LRRTM4*) and rs11750158 (near GFPT2) (p_interaction_ for sex=0.001 and 0.011, respectively).

**CONCLUSIONS:**

Habitual consumption of legumes shows protective impacts against type 2 diabetes, although these benefits vary by sex. Non-soy legumes provide health advantages for both men and women, whereas soy consumption seems to be beneficial exclusively for women.

## GRAPHICAL ABSTRACT

[Fig f1-epih-46-e2024083]

## Key Message

Our findings showed that while legume consumption was protective against type 2 diabetes, beans and peanuts demonstrated health benefits in both sexes, whereas soy’s protective benefits were observed only in women. Soy consumption was observed to have different effects between men and women, suggesting that the health impact of soy intake may differ by sex.

## INTRODUCTION

Legumes, including soybeans, beans, and peas, are vital plant-based protein sources. They are low-glycemic index foods, high in dietary fiber, and rich in health-beneficial bioactive compounds such as isoflavones [1,2]. Although commonly categorized as nuts, peanuts are botanically classified as legumes and are noted for their high antioxidant properties, including polyphenols [1].

Legumes may play a significant role in preventing cardiometabolic diseases, such as type 2 diabetes [2]. However, a recent systematic review and meta-analysis of prospective cohort studies found no association between total legume or soy intake and the risk of type 2 diabetes, with inconsistent results across different populations [3]. This variation may stem from differences in diabetes risk association among legume subtypes [3] and the predominant types of legumes consumed by different populations [4]. Notably, the review highlighted an inverse association primarily in studies conducted in Asia, where there is a relatively high soy intake. Additionally, the relationship between soy intake and cardiometabolic diseases, including type 2 diabetes, appears to vary by sex in Asian populations, showing an inverse association in women but a null or positive association in men [5-12]. However, previous studies have not adequately considered sex-specific non-genetic and genetic factors that could influence the risk of type 2 diabetes beyond legume consumption. These factors include non-genetic elements such as education level, physical activity, obesity indices, smoking status, metabolic syndrome, and impaired fasting glucose, as well as genetic factors [13,14].

The primary aim of this study was to investigate the sex-specific associations between the cumulative intake of dietary legume subtypes—including soy, beans, and peanuts—and the risk of type 2 diabetes incidence among adults aged 40 years or older in a rural cohort (CArdioVascular Disease Association Study, CAVAS). Additionally, to determine whether the observed sex-specificity could be attributed to factors other than legumes, we evaluated these associations within the same strata of non-genetic and genetic sexspecific type 2 diabetes risk factors.

## MATERIALS AND METHODS

### Study design and population

We analyzed data from 3 community cohorts aimed at preventing cardiovascular disease: the Multi-Rural Communities Cohort (MRCohort), which includes 3 rural areas (Yangpyeong, Namwon, and Goryeong); the Atherosclerosis Risk of Rural Areas in the Korean General Population (ARIRANG), encompassing 2 rural areas (Wonju and Pyeongchang); and the Kangwha cohort, another rural area. These 3 cohorts have been participating in the Korean Genome and Epidemiology Study (KoGES) since 2005. In 2008, these cohorts were consolidated into the CAVAS, and they have since been surveyed using a standardized protocol [15]. Due to the many similarities in the survey items of the KoGES, the survey protocols were already quite similar even before this unification.

A total of 19,546 participants from the CAVAS, all aged 40 years or older and free of cardiovascular disease and cancer, were enrolled between January 2005 and December 2011. Written informed consent was obtained from all participants, who were asked to revisit the research center every 2 years to 4 years for health examinations (median interval between visits during 2007-2017, 2.3 to 3.5 years). During these visits, participants completed questionnaires that provided updates on their medical history, lifestyle factors, and occurrence of cardiometabolic diseases, such as diabetes. The follow-up rate for participants who revisited more than once was 78.2%.

Participants who reported the use of any anti-diabetic drugs or insulin in the baseline survey, or whose fasting blood glucose (FBG) level at baseline was ≥ 126 mg/dL (7.0 mmol/L; n= 2,156) were excluded. We also excluded participants who: (1) left more than 10 items blank on the food frequency questionnaire (FFQ); (2) had implausible energy intake (either ≥ 99.5th or ≤ 0.5th percentile of total energy intake; n= 284); and (3) had missing data on key covariates such as education level, regular exercise, smoking status, and/or alcohol consumption (n= 440). Consequently, 16,666 participants (6,162 men and 10,504 women) were included in the final analysis.

For the entire single nucleotide polymorphism (SNP)-sex interaction analysis, a total of 8,302 participants were recruited from the MRCohort. Following standard quality control measures, which included call rate, mismatch with previous genotype, and discrepancies between genotypic and phenotypic data, 7,109 participants were eligible for inclusion in the interaction analysis (Supplementary Material 1).

### Assessment of dietary intake

Comprehensive health examinations were conducted using standard protocols to address the limitations inherent in multicenter studies. Interviewers and measurers were trained by the same coordinators using videos and hands-on training.

Well-trained interviewers collected dietary data through faceto-face interviews, using a validated FFQ that included 106 food items with 9 frequency categories ranging from “never or rarely” to “3 times/day” and 3 portion sizes specified for each item [16]. Legume consumption was assessed using 4 specific items from the FFQ: (1) soybean and soy products (tofu, fermented soybean paste, soybeans cooked in soy sauce, and soy milk); (2) beans (mung bean and green bean); (3) peanuts; and (4) peas. Although peas were accounted for in the total legume intake, they were not analyzed as a separate subtype in the present study due to their very low consumption levels.

Dietary legume consumption was then calculated as the sum of these items, derived from the 106 food items listed in the FFQ and incorporating the 475 underlying recipes that comprised these food items. We calculated the daily intake of each legume in grams by multiplying the weighted frequency (per day) by the weighted portion size (in grams). Other nutrients were calculated using the 2011 nutrient database of the Korean Nutrition Society, based on the seventh edition of the Korean Food Composition Table [17].

Our study employed FFQ data collected at 3 different time points. To more accurately reflect long-term dietary consumption and reduce within-person variation [18], we calculated the cumulative average intake of legumes. This was done by averaging its intake at baseline and at subsequent examinations, up until the endpoint was reached or the participants were censored. The average number of FFQs used to estimate the cumulative average consumption of dietary factors was 2.04, ranging from 1.00 to 3.00.

### Ascertainment of diabetes

At each visit, participants reported whether they had been diagnosed with type 2 diabetes by a physician and whether they started taking anti-diabetic drugs and/or insulin. For those who did not have type 2 diabetes at the baseline survey, we defined a new case as follows: (1) a participant who, after being newly diagnosed by a physician, began treatment with oral medication or insulin (with the reported date of diagnosis considered as the date of onset, or if this date was missing, the median date between the previous and current examinations was used); or (2) those who had an FBG level of ≥ 126 mg/dL (7.0 mmol/L) during the health examination of the revisit, in accordance with the American Diabetes Association’s recommendations [19].

### General characteristics, anthropometrics, and biochemical measurements

Data on demographics (sex, age, and educational level), lifestyle (regular exercise, smoking status, alcohol consumption, and drinking status), and medical history were collected using structured questionnaires. Height was measured with a stadiometer to the nearest 0.1 cm, and weight was assessed with a metric scale to the nearest 0.1 kg, with participants wearing light clothing and no shoes. Waist circumference (WC) was measured at the midpoint between the lowest rib margin and the iliac crest. Systolic blood pressure and diastolic blood pressure were measured in the sitting position after 5 minutes of rest with a standard mercury sphygmomanometer in the MRCohort and ARIRANG (Baumanometer; WA Baum Co. Inc., Copiague, NY, USA, or CK-101; Spirit Medical Co., New Taipei City, Taiwan) and an automatic sphygmomanometer (Dinamap 1846 SX/P or Dinamap CARESCAPE V100; GE Healthcare, Waukesha, WI, USA) in the Kangwha cohort. Blood pressure (BP) was measured twice; if the readings differed by 5 mmHg or more, up to 3 additional measurements were taken. The arithmetic mean of these readings was used for further analyses. As an exception, more than half of the baseline BP measurements in the ARIRANG cohort were taken only once (56.2%) before inclusion in the CAVAS; in these cases, a single reading was used. Blood samples were collected after a fasting period of at least 8 hours, and serum concentrations of FBG, triacylglycerol, and high-density lipoprotein cholesterol were quantified using an ADVIA 1650 Automated Analyzer (Siemens Healthcare Diagnostics, Tarrytown, NY, USA).

### Genotype data

Genotype data in the MRCohort were generated using the Korea Biobank Array (KoreanChip), designed by the Center for Genome Science at the Korea National Institute of Health. A detailed description of the criteria and procedures of the genotype quality control is provided elsewhere [20]. Briefly, sample quality control involved filtering of the genome data by discarding samples with a low call rate (< 99%), excessive heterozygosity, cryptic first-degree relatives, and sex inconsistencies. Low-quality SNPs, including off-target variants and those categorized by SNPolisher, along with SNPs having a Hardy-Weinberg equilibrium p-value less than 1.0× 10^-6^ and genotype call rates less than 95%, were excluded during SNP quality control. SNP imputation was performed using IMPUTE v2 [21] and the 1000 Genomes Project Phase 3 reference panel. To increase the statistical power of our study in detecting robust SNP-sex interactions, all SNPs were required to have a minor allele frequency (MAF) greater than 0.2 [22]. A total of 2,048,781 SNPs were identified.

### Statistical analysis

Daily legume intake was categorized into quartiles for soy and tertiles for beans and peanuts. The person-time of follow-up for each participant was calculated from the date of enrollment until the date of type 2 diabetes diagnosis. For participants lost to follow-up, half the median follow-up time of those who were successfully followed was assigned as their follow-up time, assuming that censoring occurs uniformly throughout the interval between visits [23].

Descriptive data are presented as mean± standard deviation for continuous variables and as numbers (n) and percentages (%) for categorical variables. To estimate the incidence rate ratios (IRRs) and 95% confidence intervals (CIs), we employed a modified Poisson regression model with a robust error estimator [24,25]. The primary analyses involved fitting 3 models: (1) an age-adjusted model; (2) a multivariable adjusted model (including age [years]; educational level [≥ 12 years, yes or no]; regular exercise [≥ 3 times/wk and ≥ 30 min/session)]; smoking status [non-smoking, former, or current]; alcohol consumption [g/day]; body mass index [BMI, kg/m^2^]; and total energy intake [kcal/day]); and (3) a calculation of the modified Diet Quality Index-International (DQI-I) score as a dietary covariate to reflect dietary quality. It assessed 4 key aspects: variety (overall food group variety and within-group variety for protein sources), adequacy (vegetables, fruits, grain fiber, protein, iron, calcium, and vitamin C), moderation (total fat, saturated fat, cholesterol, sodium, and empty calories), and overall balance (macronutrient ratio, fatty acid ratio). Scores were assigned to specific components within each category and then summed to obtain a total DQI-I score ranging from 0 to 100, with higher scores indicating better overall diet quality [26]. These were used to assess whether overall diet quality is a potential mediator or confounder of the association between legume consumption and type 2 diabetes risk. Regarding the tests of linear trends, the median values of legume consumption categories were treated as continuous variables, and the level of significance was set at 0.05.

To assess potential interactions between sex and the intake of legume subtypes, the cross-product term of sex and sex-specific quartile (or tertile) of legume consumption was introduced into to the multivariable model, and Wald tests were used for the statistical testing of multiplicative interaction. To rule out the influence of non-genetic and genetic risk factors other than legume consumption that might exhibit sex-specific effects, we conducted further interaction analyses between sex and legumes within the same strata of factors previously identified as sex-specific in the development of type 2 diabetes [13,14]. Initially, we considered the following non-genetic, sex-specific risk factors: higher education (yes/no), regular exercise (yes/no), obesity indices (BMI ≥ 23/< 23 kg/m^2^, WC ≥ 90/< 90 cm for men and ≥ 85/< 85 cm for women), smoking status (current/non-smoker), metabolic syndrome (≥ 3/< 3 components), and impaired fasting glucose (< 100/≥ 100 mg/dL). Secondly, we included sex-specific SNPs that were previously reported, as well as novel SNPs identified in this study. Seven SNPs—rs6275 (*DRD2/ANKK*), rs659366 (*UCP2*), rs2071746 (*HMOX1*), rs1800497 (*DRD2/ANKK1*), rs1799883 (*FABP2*), rs755622 (*MIF*), and rs7798471 (*ZNF12*)—were used as previously reported candidate SNPs [13,14], as listed in Supplementary Material 2. Novel SNPs showing significant interaction with sex in the present study were considered as genetic risk factors. However, variants that did not show a strong marginal association with type 2 diabetes due to interaction with legume consumption could have been missed. We selected novel SNPs from a pool of 2,048,781 SNPs (MAF > 0.2) using the following 2-step approach: (1) as a screening step, a joint test (2 degrees of freedom [df] test of the null hypotheses β_SNP_= 0 and β_INT_ = 0) was conducted in an age-adjusted model and SNPs with p_Joint_ < 1.0× 10^-5^ (rather than p_Joint_ < 1.0× 10^-8^) were selected [27], and (2) as the second step, we conducted a 1 df interaction analysis with sex (1 df test of the interaction product term) for SNPs selected in the screening step, and linkage disequilibrium-based clumping analyses were conducted to avoid testing correlated SNPs at the significant threshold of p_interaction_<1.0×10-5. R plugins in the PLINK toolset [28] were utilized. Each SNP was analyzed under the additive model (0, 1, or 2 copies of the minor allele as continuous). We selected regions defined as intronic or intergenic using 2 gene annotation sources, namely “UCSC Genes” and “RefSeq Genes” in the UCSC browser (http://genome.ucsc.edu).

We conducted a series of sensitivity analyses to assess the robustness of our findings. Initially, we evaluated the associations by halting the update of dietary variables upon the diagnosis of cardiovascular disease or cancer. This approach was taken because dietary changes following the onset of these conditions could obscure the relationship between diet and diabetes risk. Subsequently, we adjusted for dietary folate, calcium, and glycemic load, which were previously linked to type 2 diabetes risk in our KoGES_MRCohort study [29-31]. Additionally, we adjusted baseline FBG levels to explore the association between dietary legumes and diabetes risk under the assumption of the same FBG values at baseline. Lastly, to mitigate the potential for reverse causality bias, we excluded type 2 diabetes events that occurred within the first year from all analyses.

Analyses were carried out using SAS version 9.4 (SAS Institute Inc., Cary, NC, USA) and PLINK v1.09 [28].

### Ethics statement

The study adhered to the tenets of the Declaration of Helsinki, the protocol was approved by the appropriate institutional review boards, and all participants provided written informed consent prior to participation.

## RESULTS

The mean intake of soy, beans, and peanuts was 41.6 g/day, 1.3 g/day, and 0.6 g/day for men, and 39.3 g/day, 1.2 g/day, and 0.6 g/day for women, respectively (Supplementary Material 3). During the follow-up period (median, 5.98 years; interquartile range, 3.23 to 8.77), we accumulated 96,945 person-years of follow-up (50,287 in the MRCohort; 29,442 in the ARIRANG; and 17,216 person-years in the Kangwha cohort). A total of 945 incident cases of type 2 diabetes were documented (533 cases in the MRCohort; 252 in the ARIRANG; and 160 in the Kangwha cohort). The age-standardized baseline characteristics of the CAVAS population by quartiles of soy intake and tertiles of beans and peanuts intake are presented in Table 1. Participants in the highest quartiles or tertiles of legume consumption tended to be high school graduates, engaged in regular exercise, and consumed more alcohol compared to those in the lowest quartile (p_trend_< 0.05). Additionally, both men and women in these groups were more likely to have better diet quality and higher DQI-I scores.

The multivariable-adjusted IRRs (95% CIs) for type 2 diabetes incidence across the quartiles of legume intake are presented in Table 2. After adjusting for demographic and lifestyle factors, soy consumption was found to be inversely associated with the risk of type 2 diabetes in women (IRR for the highest vs. lowest quartile, 0.64; 95% CI, 0.50 to 0.83; p_trend_= 0.009). Further adjustment for the modified DQI-I score strengthened this association. However, no association was observed between soy consumption and type 2 diabetes incidence in men (IRR, 1.04; 95% CI, 0.75 to 1.42; p_trend_= 0.461). The association between soy consumption and type 2 diabetes risk differed significantly between men and women (p_interaction_ for sex= 0.017). In men, soy intake appeared to have a U-shaped association with type 2 diabetes risk, although this was not clearly defined. We further tested this relationship for nonlinearity using restricted cubic spline models, but the non-linearity was not statistically significant (p_non-linearity_= 0.160).

Inverse associations were observed for all non-soy legumes (beans and peanuts) in both men and women, although the association between peanut consumption and men was not significant (all p_interaction_ values for sex> 0.10).

Among the 14 sex-specific non-genetic and genetic factors previously reported, no significant sex interactions were observed in the present study. These factors included higher education, regular exercise, obesity indices, smoking status, metabolic syndrome, and impaired fasting glucose, with a p_interaction_ for sex < 0.003 across 16 multiple tests using Bonferroni correction (Table 3, Supplementary Material 4). Two novel SNPs, rs10196939 (_LRRTM4_) and rs11750158 (near *GFPT2*), were identified in the genome-wide sex interaction analysis for sex-specific genetic factors (p_interaction_ for sex= 6.77× 10^-6^ and 1.59× 10^-5^, respectively; Table 3). Table 4 presents the association between soy and type 2 diabetes within the same strata of the 2 novel, sex-specific genetic factors. The sex-specific association between soy and type 2 diabetes risk remained in carriers of the minor allele, but not in those with the wild-type counterparts. Moreover, the interaction between sex and soy was antagonistic, contrasting with the unilateral association observed when these genotypes were not considered. Specifically, men who were minor allele carriers of rs10196939 and rs11750158 exhibited a higher, though not statistically significant, risk of type 2 diabetes with high soy intake (IRR, 1.57; 95% CI, 0.90 to 2.74; p_trend_= 0.010; p_interaction_ for sex= 0.001 for rs10196939; IRR, 1.41; 95% CI, 0.79 to 2.49; p_trend_= 0.054; p_interaction_ for sex= 0.011 for rs11750158), whereas a significant inverse trend was observed in women. To verify these findings across the 3 regions of the MRCohort, we examined the association between soy intake and type 2 diabetes within the same genetic strata in each region. The results were consistent, although most interactions did not reach statistical significance (Supplementary Material 5).

## DISCUSSION

In the present study, the consumption of non-soy legumes, such as beans and peanuts, was found to be inversely associated with the risk of developing type 2 diabetes in both men and women. However, habitual dietary intake of soy was associated with type 2 diabetes risk in women, but not in men. This sex-specific association between soy intake and type 2 diabetes risk appeared to be antagonistic among minor allele carriers after stratification of sex-specific SNPs (rs10196939 and rs11750158) associated with type 2 diabetes risk. While the inverse association persisted in women, a positive trend was observed in men. To our knowledge, these are the first prospective findings on the sex-specific associations between legume subtypes and type 2 diabetes.

Previous cross-sectional [6,10] and prospective studies conducted in Asia [7,11], where soy is predominantly consumed, do not support the notion that habitual dietary soy consumption offers protection against diabetes in men, unlike other non-soy legumes. For example, a Chinese study demonstrated that habitual intake of soy protein was sex-specific and associated with hyperglycemia in middle-aged and older adults, with a positive association observed only in men [6]. Similarly, India’s Third National Family Health Survey showed that women who consumed legumes daily or weekly had a significantly lower prevalence of type 2 diabetes compared to those who did not consume legumes; this association was not observed in men [10]. Additionally, 2 Japanese prospective studies observed an inverse association between soy food or product consumption and type 2 diabetes risk in women, but not in men (median intake ≥ 117.3 g/day) [7,11]. However, this sex difference may stem from differences in the types of soy foods consumed by men versus women, such as soymilk, which has been identified as a potential risk factor for diabetes in the Singapore Chinese Health Study [32]. In our additional analysis that excluded soymilk, the sex difference in the association between soy consumption and type 2 diabetes risk was retained (IRR for the highest vs. lowest quartile in men and women, respectively: IRR, 1.19; 95% CI, 0.87 to 1.64; p_trend_= 0.141; IRR, 0.69; 95% CI, 0.52 to 0.90; p_trend_= 0.055). This association was primarily observed in Asian populations but was not restricted to them, despite scant evidence of this association in Western populations [33]. Additionally, there was a sex-specific association between dietary soy foods or isoflavones with type 2 diabetes risk, being inversely associated in American women and not significant in American men [34].

This discrepancy may stem from the estrogenic properties of isoflavones, which are particularly abundant in soy compared to other legumes. Theoretically, the estrogen-like activity of soy isoflavones could inhibit enzymes involved in hormone metabolism, such as 17β-hydroxysteroid oxidoreductase or cytochrome P450 isozymes, potentially leading to reduced testosterone levels [35,36]. This hypothesis is supported by experimental and observational studies that have found an inverse relationship between soy consumption and endogenous testosterone levels in men [37-39], although this finding is not consistent in all trial studies [40]. In some studies, soy intake was shown to increase the level of sex hormone-binding globulin [41,42], which could further decrease free testosterone levels. While this may be beneficial for women, it could be detrimental for men, as lower testosterone levels are associated with an increased risk of type 2 diabetes in men but a decreased risk in women [43]. Overall, the observed null association between soy consumption and the risk of type 2 diabetes in men may be due to the masking of the anti-diabetic effects of beneficial components such as plant protein, fiber, and antioxidants found in soy legumes. These benefits could be offset by the potentially adverse effects of soy isoflavones on hormonal metabolism.

Among the factors previously reported as sex-specific for diabetes [13], no significant interaction with sex was observed in the current study. However, a genome-wide sex interaction analysis identified 2 novel SNPs, which were observed to be sex-specific risk factors for the development of type 2 diabetes, showed a positive trend in the association between soy consumption and type 2 diabetes risk in men who were minor allele carriers of rs10196939 (in *L*RRTM4**) and rs11750158 (near *GFPT2*).

*L*RRTM4**, a member of the four-gene L*RRTM* gene family, plays a role in regulating the development and strength of glutamatergic synapses [44]. Gonadal hormones, such as estrogen and testosterone, have been shown to increase the formation and transmission of glutamatergic synapses, albeit through distinct mechanisms in men and women [45,46]. A gene-sequencing study identified sex-specific variants in *L*RRTM4**, suggesting that these loci may impact the function of neurons in the central nervous system in a sex-specific manner [46]. *GFPT2*, which is encoded by the *GFPT2* gene, acts as a rate-limiting enzyme in the hexosamine biosynthetic pathway, which not only controls the flux of glucose within the hexosamine pathway but also regulates the N-linked and O-linked glycosylation of proteins [47]. The *GFPT2* gene was previously implicated in insulin resistance in type 2 diabetes [48]. However, to date, research on sex differences in this context has been limited. While there is no direct evidence linking dietary soy, *L*RRTM4**, or *GFPT2* with type 2 diabetes risk, the potential for such a connection exists, warranting further investigation.

Our findings on the relationship between various legume subtypes and type 2 diabetes remain robust across several sensitivity analyses under the following conditions: (1) discontinuing diet updates and censoring cases of cardiovascular disease or cancer that emerged during the follow-up period; (2) further adjusting for dietary folate, calcium, and glycemic load, all of which have been previously linked to diabetes risk in our MRCohort; (3) adjusting for baseline FBG; and (4) excluding incident cases from the first year of follow-up (data not shown).

There are several limitations to the interpretation of our findings. First, the loss to follow-up may have introduced biases. Although we observed small differences in legume consumption patterns among participants with different follow-up statuses—specifically, more beans consumed by men and more peanuts by both sexes—our sensitivity analysis, which was restricted to those who completed both the baseline and one or two follow-up surveys, yielded results consistent with our main findings. While this approach does not entirely eliminate the possibility of follow-up bias, the consistency between the main and sensitivity analyses lends support to the robustness of our results.

Second, the substantial reduction in diabetes incidence observed with relatively small differences in non-soy legume intake is noteworthy. This could potentially indicate that: (1) even modest increases in legume intake could have substantial metabolic benefits; (2) the association with type 2 diabetes might be influenced by dietary patterns associated with legume intake rather than by the legumes themselves, as higher legume consumption is often linked with beneficial foods or nutrients that reduce type 2 diabetes risk, such as dietary folate, calcium, and low glycemic index foods [29-31,49]. Therefore, we cannot completely dismiss the possibility that legume consumers may engage in other health-promoting behaviors that could influence the observed association; and (3) the FFQ is designed to capture relative intake rather than absolute intake, necessitating caution when interpreting the absolute effect size. Third, we cannot recommend specific absolute intake values of non-soy legumes for men and women, or soy for women, for the prevention of type 2 diabetes because their consumption was estimated using the FFQ, which does not measure absolute dietary intake. Fourth, the CAVAS is a multicenter cohort study. Despite implementing quality control procedures— such as training by the epidemiologic data center, site visits, observations, evaluations, and feedback—to minimize differences in data quality across centers, we also conducted separate analyses for each cohort and then pooled the results to confirm the robustness of our findings. This analysis produced findings very similar to our primary analysis. Specifically, for soy intake, the pooled results from the cohort-specific analyses showed that, in men, the IRR for quartile 4 compared with quartile 1 was 1.08 (95% CI, 0.78 to 1.49; p_trend_= 0.393), and in women, the IRR for quartile 4 compared with quartile 1 was 0.59 (95% CI, 0.45 to 0.77; p_trend_= 0.001). Detailed results are presented in Supplementary Material 6. Fifth, due to the exploratory nature of our gene-diet interaction analysis and the lack of a suitable replication cohort with both genetic and cumulative dietary intake data, our findings on novel SNPs should be interpreted with caution. Further studies in independent populations are necessary to validate these results and to fully elucidate the sex-specific effects of cumulative soy intake and genetic factors on type diabetes risk. Finally, although we adjusted for potential confounders, we could not rule out the residual confounding effects of unmeasured factors, such as the phytoestrogen metabolites equol, enterolactone, and enterodiol, which have higher binding affinities for hormone [36,50]. Further prospective studies using phytoestrogen biomarkers are needed to determine whether habitual legume consumption and sex hormones interact in the development of type 2 diabetes. Nevertheless, in the present study, the use of cumulative average consumption based on repeated dietary measures could improve the quality of dietary information and better reflect habitual dietary intake. Additionally, we considered various factors, including genetic variants, that may be interactive or influential.

Habitual intake of non-soy legumes is inversely associated with type 2 diabetes in both men and women, whereas dietary soy shows an inverse association with type 2 diabetes incidence only in women. These findings suggest that dietary factors may affect the development of type 2 diabetes differently in men versus women. The observed sex-specific differences in soy consumption warrant replication in future prospective studies, and additional research is needed to explore potential underlying mechanisms, such as sex hormone biomarkers.

## Figures and Tables

**Figure f1-epih-46-e2024083:**
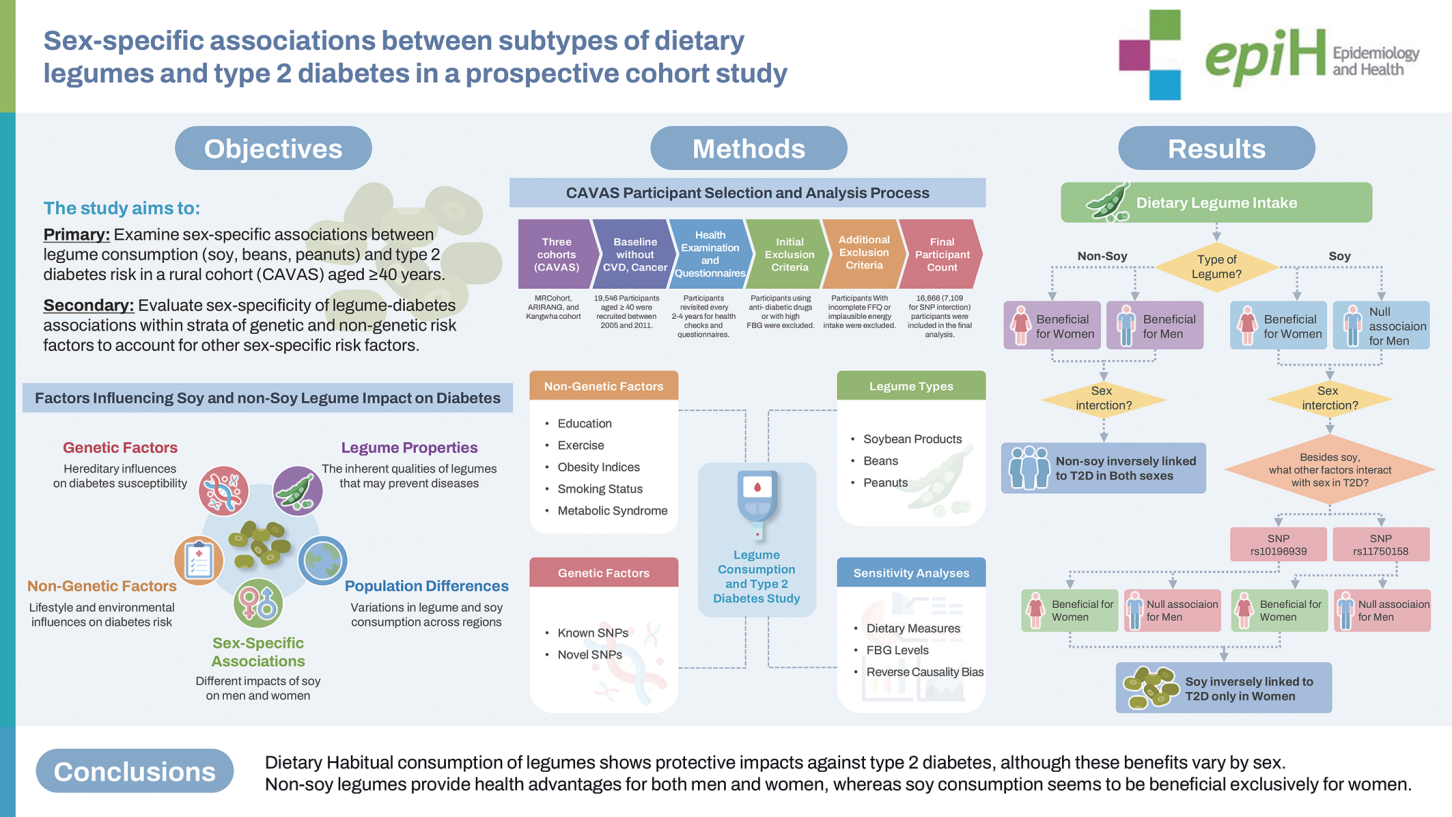


**Table 1.** Age-standardized characteristics of the study population according to quartiles (Q) or tertiles (T) of dietary legumes

Characteristics	Q of dietary soy (g/day)				p_trend_^1^	T of dietary beans (g/day)			p_trend_^1^	T of dietary peanuts (g/d)			p_trend_^1^
	Q1	Q2	Q3	Q4		T1	T2	T3		T1	T2	T3	
Men (n=6,162)													
Median intake (interquartile range)	10.0 (6.4, 13.5)	23.2 (19.8, 26.3)	38.8 (34.3, 44.9)	75.1 (60.1, 108.4)		0 (0, 0.08)	0.5 (0.3, 0.7)	2.1 (1.4, 3.6)		0 (0, 0)	0.2 (0.1, 0.2)	0.9 (0.5, 2.0)	
Age (yr)	60.5±9.9	58.9±9.5	58.1±9.3	58.7±9.7	<0.001	60.1±9.9	58.7±9.6	58.5±9.3	<0.001	59.9±10.1	58.6±9.2	58.3±9.1	<0.001
Higher education^2^	32.7	38.2	39.4	41.0	<0.001	33.1	38.8	41.5	<0.001	31.3	36.3	47.4	<0.001
Regular exercise^3^	17.6	20.7	21.7	23.8	<0.001	18.2	21.1	23.7	<0.001	15.4	20.0	29.0	<0.001
Current smoker	34.6	35.2	35.6	37.8	0.068	40.3	36.4	31.2	<0.001	39.0	36.2	31.5	<0.001
Alcohol consumption (g/day)	20.2±36.9	23.3±44.1	21.7±38.0	26.1±43.2	<0.001	27.7±45.2	23.8±43.1	17.2±32.2	<0.001	22.3±38.6	21.5±40.6	24.5±43.0	0.041
Body mass index (kg/m^2^)	23.8±2.9	24.1±2.8	24.2±2.9	24.1±2.9	0.106	23.9±2.9	24.1±2.8	24.2±2.9	0.002	23.9±2.9	24.0±2.9	24.3±2.8	<0.001
Total energy intake (kcal/day)	1,468±330	1,620±356	1,723±368	1,915±453	<0.001	1,547±385	1,639±363	1,862±422	<0.001	1,599±403	1,645±353	1,821±428	<0.001
Modified Diet Quality Index^4^	62.1±6.1	65.2±5.8	66.9±5.6	69.2±5.6	<0.001	63.8±6.4	65.6±6.0	68.1±5.9	<0.001	64.1±6.3	65.5±5.8	68.4±5.8	<0.001
Women (n=10,504)													
Median intake (interquartile range)	8.9 (5.2, 11.9)	20.5 (17.7, 23.3)	35.6 (31.0, 41.0)	73.2 (57.3, 106.0)		0 (0, 0.08)	0.5 (0.4, 0.7)	2.1 (1.4, 3.3)		0 (0, 0)	0.2 (0.1, 0.2)	0.9 (0.5, 2.3)	
Age (yr)	60.6±10.0	57.6±9.5	56.2±9.3	56.5±9.7	<0.001	60.4±10.0	57.5±9.5	55.2±9.2	<0.001	59.7±10.1	57.0±9.5	55.1±8.7	<0.001
Higher education^2^	17.0	22.6	23.2	27.5	<0.001	18.1	23.0	26.9	<0.001	15.6	21.9	31.9	<0.001
Regular exercise^3^	18.0	20.9	22.4	26.6	<0.001	18.9	23.6	23.7	<0.001	16.7	22.1	29.2	<0.001
Current smoker	3.8	2.1	3.0	2.0	0.004	3.7	2.2	2.6	0.060	3.3	1.7	2.6	0.134
Alcohol consumption (g/day)	1.68±6.9	1.84±6.8	1.78±6.5	2.25±11.0	0.025	2.45±8.2	1.74±7.5	1.68±9.0	<0.001	1.89±7.1	2.04±8.3	1.89±9.3	0.506
Body mass index (kg/m^2^)	24.4±3.2	24.4±3.1	24.5±3.2	24.4±3.2	0.860	24.4±3.2	24.5±3.1	24.4±3.2	0.968	24.6±3.3	24.4±3.1	24.3±3.1	<0.001
Total energy intake (kcal/day)	1,299±326	1,422±321	1,525±344	1,699±437	<0.001	1,354±347	1,463±339	1,649±420	<0.001	1,418±376	1,474±357	1,605±399	<0.001
Modified Diet Quality Index^4^	62.8±6.8	66.0±6.2	68.2±6.0	70.6±6.0	<0.001	64.5±7.0	67.1±6.5	69.3±6.2	<0.001	65.0±6.8	66.8±6.3	69.9±6.1	<0.001

Values are presented as mean±standard deviation for continuous variables or percentage for categorical variables.

1 By imputing the median value of each category and treating it as a continuous variable by general linear modelling.

2 Higher education level (≥12 years of education).

3 Regular exercise (≥3 times/wk and ≥30 min/session).

4 Legumes are excluded from the variety category of the original version Modified Diet Quality Index-International.

**Table 2.** Type 2 diabetes mellitus according to dietary legumes

Model^1^	Dietary legumes (g/day)										p_interaction_ for sex^3^
	Men				p_trend_^2^	Women				p_trend_^2^	
Soy (soybeans and soy products)	Q1	Q2	Q3	Q4		Q1	Q2	Q3	Q4		
Median intake (Min-Max) (g/day)	10 (0-17)	23 (17-30)	39 (30-51)	75 (51-519)		9 (0-15)	21 (15-27)	36 (27-48)	73 (48-1310)		
No. of cases/person-years	90/7,936	90/9,195	118/9,579	112/9,122		172/13,721	125/15,501	112/16,102	126/15,789		
Age-adjusted	1.00	0.87 (0.65, 1.16)	1.10 (0.84, 1.44)	1.09 (0.83, 1.44)	0.247	1.00	0.68 (0.54, 0.86)	0.60 (0.47, 0.77)	0.68 (0.54, 0.86)	0.016	0.003
Multivariable	1.00	0.83 (0.62, 1.11)	0.98 (0.74, 1.30)	0.99 (0.73, 1.34)	0.635	1.00	0.68 (0.54, 0.86)	0.57 (0.45, 0.73)	0.64 (0.50, 0.83)	0.009	0.018
Multivariable+Diet quality^4^	1.00	0.85 (0.63, 1.14)	1.02 (0.76, 1.35)	1.04 (0.75, 1.42)	0.461	1.00	0.66 (0.52, 0.84)	0.55 (0.43, 0.71)	0.61 (0.48, 0.80)	0.007	0.017
Beans	T1	T2	T3			T1	T2	T3			
Median intake (Min-Max) (g/day)	0 (0-0.2)	0.5 (0.2–1.0)	2.1 (1.0-80.6)			0 (0-0.2)	0.5 (0.2-1.0)	2.0 (1.0-53.6)			
No. of cases/person-years	147/10,528	126/12,693	137/12,609			222/17,784	166/22,057	147/21,273			
Age-adjusted	1.00	0.71 (0.56, 0.91)	0.78 (0.62, 0.99)		0.190	1.00	0.63 (0.52, 0.77)	0.61 (0.49, 0.75)		<0.001	0.164
Multivariable	1.00	0.68 (0.54, 0.86)	0.69 (0.54, 0.89)		0.035	1.00	0.62 (0.51, 0.76)	0.57 (0.45, 0.72)		<0.001	0.414
Multivariable+Diet quality^4^	1.00	0.68 (0.54, 0.87)	0.70 (0.54, 0.90)		0.042	1.00	0.62 (0.50, 0.76)	0.56 (0.45, 0.71)		<0.001	0.409
Peanuts	T1	T2	T3			T1	T2	T3			
Median intake (Min-Max) (g/day)	0 (0-0)	0 (0-0.1)	0.1 (0.1-1.5)			0 (0-0)	0.2 (0.1-0.2)	0.9 (0.2-33.8)			
No. of cases/person-years	192/13,729	70/9,519	148/12,583			293/26,366	81/12,322	161/22,426			
Age-adjusted	1.00	0.53 (0.40, 0.69)	0.85 (0.68, 1.05)		0.716	1.00	0.62 (0.48, 0.79)	0.70 (0.58, 0.86)		0.006	0.138
Multivariable	1.00	0.52 (0.40, 0.69)	0.80 (0.64, 1.00)		0.395	1.00	0.65 (0.51, 0.83)	0.73 (0.59, 0.90)		0.025	0.268
Multivariable+Diet quality^4^	1.00	0.53 (0.40, 0.69)	0.81 (0.64, 1.02)		0.503	1.00	0.64 (0.51, 0.82)	0.72 (0.58, 0.89)		0.020	0.268

Values are presented as incidence rate ratio (95% confidence interval). Min, minimum; Max, maximum; Q, quartile; T, tertiles.

1 The multivariable model was adjusted for age (years), higher education level (≥12 years of education), regular exercise (≥3 times/wk and ≥30 min/session), smoking status (never/former/current), alcohol consumption (g/day), body mass index (kg/m^2^), and total energy intake (kcal/day) in men and women.

2 By imputing the median value of each Q for soy and T for beans and peanuts and treating it as a continuous variable using a modified Poisson regression with a robust error estimator.

3 By including the cross-product term of sex and sex-specific Q (or T) of legumes in the Poisson regression model to test for multiplicative interaction (Wald statistic).

4 Modified Diet Quality Index-International score.

**Table 3.** Genetic loci related to type 2 diabetes mellitus with sex differences in the sub-population (MRCohort study)

SNP (nearest gene)^1^	Chromosomallocation^2^	Type of variant	Major>minor	Minor allele frequency	Gene*sex p_interaction_ in age-adjusted model	Gene*sex p_interaction_ in multivariable model	p_joint_ test (2 df) in age-adjusted model^3^	p_interaction_ (1 df) in age-adjusted model^4^	p_interaction_ (1 df) in multivariable model^5^
Reported genetic loci with sex differences									
rs6275 (*DRD2/ANKK1*)	11q23.2	synonymous	C>T	0.49	0.7469	0.8700	0.8569	0.5884	0.7138
rs659366 (*UCP2*)	11q13.4	upstream	C>T	0.48	0.3667	0.2984	0.4298	0.1989	0.1784
rs2071746 (*HMOX1*)	22q12.3	upstream	T>A	0.46	0.9034	0.8904	0.8782	0.7316	0.0866
rs1800497 (*DRD2/ANKK1*)	11q23.2	missense	C>T	0.41	0.2981	0.2397	0.1808	0.0660	0.1263
rs1799883 (*FABP2*)	4q26	missense	A>G	0.34	0.7731	0.7245	0.8253	0.5426	0.4571
rs7798471 (*ZNF12*)	7p22.1	intron	C>T	0.24	0.3017	0.2175	0.9225	0.9857	0.6626
rs755622 (*MIF*)	22q11.23	upstream	C>G	0.22	0.7522	0.8129	0.5662	0.2879	0.3657
Novel genetic loci exerting genome-wide sex-interaction in MRCohort^6^									
rs10196939 (*LRRTM4*)	2p12	intron	A>G	0.41	8.84×10^-4^	2.30×10^-3^	8.35×10^-6^	2.26×10^-6^	6.77×10^-6^
rs11750158 (near *GFPT2*)	5q35.3	intergenic	G>A	0.33	6.80×10^-5^	3.52×10^-5^	2.16×10^-6^	2.98×10^-5^	1.59×10^-5^

MRCohort, Multi-Rural Communities Cohort; SNP, single nucleotide polymorphism; df, degrees of freedom; NCBI, National Center for Biotechnology Information.

1 SNP identifier based on NCBI dbSNP with minor allele frequency greater than 0.2.

2 Chromosomal location based on NCBI Human Genome Build 37 coordinates.

3 Joint interaction analyses were the [SNP+SNP*sex] test with 2 df adjusted for age (years); The test examines the significance of the SNP effect (additive) and its interaction with sex.

4 Interaction analyses were [SNP*sex] test with 1 df adjusted for age (years); The test examines the interaction between the SNP effect (additive) and sex.

5 Interaction analyses were [SNP*sex] test with 1 df adjusted for age (years), higher education level (≥12 years of education), regular exercise (≥3 times/wk and ≥30 min/session), smoking status (never/former/current), alcohol consumption (g/day), body mass index (kg/m^2^), total energy intake (kcal/day), and modified Diet Quality Index-International score; The test examines the interaction between the SNP effect (additive) and sex.

6 SNPs with minor allele frequency greater than 0.2 and p-joint interaction less than 1.00e-05 in age-adjusted model.

**Table 4.** Type 2 diabetes mellitus according to dietary soy consumption and genotype of sex difference in MRCohort

Model^1^	Dietary soy consumption (g/day)				p_trend_^2^	p_interaction_^3^
	Q1	Q2	Q3	Q4		
rs10196939 (*LRRTM4*)						
AA, wild-type						
Men						
No. of cases/person-years	15/1,329	16/1,516	13/1,392	11/1,365		0.961
Multivariable	1.00 (reference)	0.91 (0.45, 1.83)	0.67 (0.28, 1.57)	0.74 (0.31, 1.78)	0.523	
Women						
No. of cases/person-years	37/2,038	31/2,256	24/2,665	26/2,523		
Multivariable	1.00 (reference)	0.82 (0.50, 1.35)	0.52 (0.29, 0.93)	0.59 (0.33, 1.06)	0.113	
AG and GG						
Men						
No. of cases/person-years	23/1,980	18/2,364	43/2,581	46/2,492		0.001
Multivariable	1.00 (reference)	0.63 (0.34, 1.15)	1.33 (0.78, 2.27)	1.57 (0.90, 2.74)	0.010	
Women						
No. of cases/person-years	58/4,013	33/4,609	33/4,653	40/4,838		
Multivariable	1.00 (reference)	0.51 (0.33, 0.80)	0.45 (0.29, 0.71)	0.49 (0.31, 0.77)	0.037	
rs11750158 (near *GFPT2*)						
GG, wild-type						
Men						
No. of cases/person-years	13/1,518	9/1,701	16/1,789	16/1,731		0.274
Multivariable	1.00 (reference)	0.64 (0.39, 1.05)	0.53 (0.31, 0.91)	0.57 (0.34, 0.98)	0.542	
Women						
No. of cases/person-years	43/2,409	29/2,953	26/3,113	29/3,159		
Multivariable	1.00 (reference)	0.64 (0.39, 1.05)	0.53 (0.31, 0.91)	0.57 (0.34, 0.98)	0.135	
GA and AA						
Men						
No. of cases/person-years	24/1,721	21/2,041	40/2,104	40/2,069		0.011
Multivariable	1.00 (reference)	0.71 (0.39, 1.28)	1.18 (0.69, 2.04)	1.41 (0.79, 2.49)	0.054	
Women						
No. of cases/person-years	48/3,416	34/3,654	29/4,082	36/4,025		
Multivariable	1.00 (reference)	0.64 (0.40, 1.02)	0.44 (0.27, 0.72)	0.51 (0.30, 0.84)	0.043	

Values are presented as incidence rate ratio (95% confidence interval). MRCohort, Multi-Rural Communities Cohort; Q, quartile.

1 The multivariable model was adjusted for age (years), higher education level (≥12 years of education), regular exercise (≥3 times/wk and ≥30 min/session), current smoking status (yes or no), alcohol consumption (g/day), body mass index (kg/m^2^), total energy intake (kcal/day), and modified Diet Quality Index-International in men and women.

2 By imputing the median value of each Q and treating it as a continuous variable using a modified Poisson regression with a robust error estimator.

3 By including the cross-product term of Q of soy consumption and sex in the Poisson regression model to test for multiplicative interaction (Wald statistic).

## Supplementary materials

Supplementary Material 1. Flow chart of study participants

Supplementary Material 2. Previously reported sex-specific SNPs associated with diabetes

Supplementary Material 3. General characteristics of the study population

Supplementary Material 4. Analyses of the interaction between sex and previously reported sex-specific non-genetic factors for the risk of type 2 diabetes

Supplementary Material 5. Incidence rate ratio (IRR) and 95% confidence intervals (CI) of type 2 diabetes mellitus (T2DM) according to dietary soy consumption and genotype of sex difference in each of the three regions in MRCohort

Supplementary Material 6. Incidence rate ratio (IRR) and 95% confidence intervals (CI) of type 2 diabetes by dietary legumes in the three cohorts
