# Supplementary material for: Sex-specific associations between dietary legume subtypes and type 2 diabetes in a prospective cohort study
Source: Epidemiol Health. 2024 Oct 17;46:e2024083. doi: 10.4178/epih.e2024083 (PMC11832243; doi:10.4178/epih.e2024083)
Supplement: Supplementary Material 1. — Flow chart of study participants [file epih-46-e2024083-Supplementary-1.docx]

**Supplementary Material 1.** Flow chart of study participants

**CAVAS Study** (2005-2017) consisting

MRCohort, ARIRANG, Kangwha cohort

**19,546 participants** without cardiovascular disease and/or any cancers at baseline

After standard quality control

(call rate, mismatch with previous genotype, and genotypic and phenotypic mismatch)

Participants who reported the use of any anti-diabetic drugs or insulin in the baseline survey, or whose fasting blood glucose (FBG) level at baseline was ≥ 126 mg/dL (7.0 mmol/L) (n=2,156) were excluded.

We also excluded the following participants:

(1) those who left **>**10 items blank on the food frequency questionnaire (FFQ);

(2) those who had implausible energy intake (≥ 99.5^th^ percentile or ≤ 0.5^th^ percentile of total energy intake) (n=284);

(3) those who had missing data of important covariates, such as education level, regular exercise, smoking status, and/or alcohol consumption (n=440).

**7,109 participants form MRCohot**

were included in the whole single nucleotide polymorphism-sex interaction analysis

**16,666 participants**

(6,162 men and 10,504 women) were included in the final analysis
