# Supplementary material for: Sex-specific associations between dietary legume subtypes and type 2 diabetes in a prospective cohort study
Source: Epidemiol Health. 2024 Oct 17;46:e2024083. doi: 10.4178/epih.e2024083 (PMC11832243; doi:10.4178/epih.e2024083)
Supplement: Supplementary Material 2. — Previously reported sex-specific SNPs associated with diabetes [file epih-46-e2024083-Supplementary-2.docx]

**Supplementary Material 2**. SNPs previously reported sex-specific association with diabetes [13,14]

| SNP^1^ | Chromosomal  location ^2^ | Nearest gene | Gene Description | Reported Sex Differences [13] |
| --- | --- | --- | --- | --- |
| rs6275 | 11q23.2 | ***DRD2*** | Dopamine receptor whose activity is mediated by G proteins which inhibit adenylyl cyclase | Increased first-phase glucose-stimulated insulin secretion in women, but not in men |
|  |  | ***ANKK1*** | Ankyrin repeat and kinase domain containing 1, and this gene is closely linked to DRD2 gene |  |
| rs659366 | 11q13.4 | ***UCP2*** | Mitochondrial uncoupling proteins (UCP) are members of the larger family of mitochondrial anion carrier proteins (MACP) | AA genotype associated with T2DM in women but not in men |
| rs2071746 | 22q12.3 | ***HMOX1*** | Catalyzes the oxidative cleavage of heme at the alpha-methene bridge carbon, released as carbon monoxide (CO), to generate biliverdin IXalpha, while releasing the central heme iron chelate as ferrous iron. | TT genotype is associated with albuminuria in T2DM; male carriers are at higher risk for albuminuria, not female carrier |
| rs1800497 | 11q23.2 | ***DRD2*** | Dopamine receptor whose activity is mediated by G proteins which inhibit adenylyl cyclase | Increased risk for T2DM in women but not in men |
|  |  | ***ANKK1*** | Ankyrin repeat and kinase domain containing 1, and this gene is closely linked to DRD2 gene |  |
| rs1799883 | 4q26 | ***FABP2, Ala54Thr*** | It is probably involved in triglyceride-rich lipoprotein synthesis. Binds saturated long-chain fatty acids with a high affinity, but binds with a lower affinity to unsaturated long- chain fatty acids. FABP2 may also help maintain energy homeostasis by functioning as a lipid sensor. | Homozygous Thr54 variant associated with reduced risk of T2DM in women but not in men |
| rs7798471 | 7p22.1 | ***ZNF12*** | This gene is a member of the krueppel C2H2-type zinc-finger protein family and encodes a protein with eight C2H2-type zinc fingers and a KRAB domain. | Sex-specific effect on higher FI levels within the first intron of ZNF12 at rs7798471-C |
| rs755622 | 22q11.23 | ***MIF*** | Pro-inflammatory cytokine. Involved in the innate immune response to bacterial pathogens. The expression of MIF at sites of inflammation suggests a role as mediator in regulating the function of macrophages in host defense. | C allele associated with increased abdominal obesity, apolipoprotein B levels, and higher risk for development of T2DM in men |

^1^ Candidate SNPs previously reported to be sex-specifically associated with diabetes [13,14].

^2^ Chromosomal location based on NCBI Human Genome Build 37 coordinates.
