# Supplementary material for: Sex-specific associations between dietary legume subtypes and type 2 diabetes in a prospective cohort study
Source: Epidemiol Health. 2024 Oct 17;46:e2024083. doi: 10.4178/epih.e2024083 (PMC11832243; doi:10.4178/epih.e2024083)
Supplement: Supplementary Material 3. — General characteristics of the study population [file epih-46-e2024083-Supplementary-3.docx]

**Supplementary Material 3.** General characteristics of the study population

| Characteristics^1^ | Total | MEN | WOMEN |
| --- | --- | --- | --- |
| ***N*** | *16,666* | *6,162* | *10,504* |
| Age, y | 58.2 ± 9.7 | 59.1 ± 9.6 | 57.7 ± 9.8 |
| Higher education^2^, % | 28.3 | 37.8 | 22.8 |
| Regular exercise^3^, % | 21.7 | 21.0 | 22.0 |
| Smoking status, % |  |  |  |
| Never smoker | 71.2 | 29.0 | 95.9 |
| Former smoker | 13.8 | 35.2 | 1.3 |
| Current smoker | 15.0 | 35.8 | 2.8 |
| Alcohol consumption, g/d | 9.63 ± 27.7 | 22.8 ± 40.9 | 1.9 ± 8.4 |
| Body Mass Index, kg/m^2^ | 24.3 ± 3.1 | 24.1 ± 3.0 | 24.4 ± 3.2 |
| Total energy intake, kcal/d | 1,561 ± 424 | 1,683 ± 429 | 1,489 ± 405 |
| Modified Diet Quality Index-International (DQI-I)^4^ | 66.5 ± 6.9 | 65.8 ± 6.4 | 66.9 ± 7.1 |
| Habitual dietary consumption, g/d |  |  |  |
| Total legume | 42.0 ± 43.3 | 43.5 ± 41.7 | 41.1 ± 44.2 |
| Soy consumption (soybeans and soy products) | 40.1 ± 42.7 | 41.6 ± 41.0 | 39.3 ± 43.6 |
| Beans | 1.2 ± 2.4 | 1.3 ± 2.5 | 1.2 ± 2.3 |
| Peanuts | 0.6 ± 1.6 | 0.6 ± 1.6 | 0.6 ± 1.6 |

^1^ The values are expressed as Mean ± SD for continuous variables or percentage for categorical variables.

^2^ Higher education level (≥12 years of education).

^3^ Regular exercise (≥3 times/week and ≥30 min/session).

^4^ Legumes are excluded from the variety category of the original version Modified Diet Quality Index-International (DQI-I).
