# Supplementary material for: Sex-specific associations between dietary legume subtypes and type 2 diabetes in a prospective cohort study
Source: Epidemiol Health. 2024 Oct 17;46:e2024083. doi: 10.4178/epih.e2024083 (PMC11832243; doi:10.4178/epih.e2024083)
Supplement: Supplementary Material 4. — Analyses of the interaction between sex and previously reported sex-specific non-genetic factors for the risk of type 2 diabetes [file epih-46-e2024083-Supplementary-4.docx]

**Supplementary Material 4.** Analyses of the interaction between sex and previously reported sex-specific non-genetic factors for the risk of type 2 diabetes

| Non-genetic factors^1^ | Sex | | *P*-difference | *P*-interaction^4^ |
| --- | --- | --- | --- | --- |
|  | Women | Men |  |  |
| Education level |  |  |  |  |
| ≥ 12 years (n=4,722) | 1.00 | 1.21 (0.87-1.66) | 0.2583 | 0.1895 |
| < 12 years (n=11,944) | 1.00 | 1.19 (0.97-1.44) | 0.0897 |  |
| Regular exercise^2^ |  |  |  |  |
| Yes (n=3,608) | 1.00 | 1.19 (0.83-1.71) | 0.3516 | 0.3192 |
| No (n=13,058) | 1.00 | 1.20 (0.997-1.44) | 0.0530 |  |
| Body Mass Index |  |  |  |  |
| < 23 kg/m^2^ (n=5,862) | 1.00 | 1.08 (0.72-1.62) | 0.7096 | 0.5337 |
| ≥ 23 kg/m^2^ (n=10,804) | 1.00 | 1.21 (1.01-1.45) | 0.0352 |  |
| Waist circumference |  |  |  |  |
| Normal (n=10,582) | 1.00 | 1.11 (0.86-1.42) | 0.4205 | 0.7870 |
| Abdominal obesity^3^ (n=6,084) | 1.00 | 1.28 (1.03-1.60) | 0.0292 |  |
| Current smoking status |  |  |  |  |
| No (n=14,165) | 1.00 | 1.23 (1.03-1.46) | 0.0224 | 0.3731 |
| Yes (n=2,501) | 1.00 | 0.97 (0.59-1.58) | 0.8986 |  |
| Clustering of metabolic risk factors |  |  |  |  |
| < 3 MetS components (n=10,820) | 1.00 | 1.35 (1.01-1.81) | 0.0415 | 0.1559 |
| ≥ 3 MetS components (5,841) | 1.00 | 1.13 (0.92-1.39) | 0.2298 |  |
| Impaired fasting glucose |  |  |  |  |
| Fasting blood glucose < 100 mg/dL (n=12,599) | 1.00 | 1.06 (0.79-1.42) | 0.6898 | 0.0141 |
| Fasting blood glucose ≥ 100 mg/dL (n=4,067) | 1.00 | 0.85 (0.70-1.04) | 0.1078 |  |

^1^ The multivariable model was adjusted for age (years), higher education level (≥ 12 years of education), regular exercise (≥ 3 times/week and ≥ 30 minutes/session), current smoking status (yes or no), alcohol consumption (g/d), body mass index (kg/m^2^), total energy intake(kcal/d), and modified Diet Quality Index-International (DQI-I) in men and women.

^2^ Regular exercise (≥3 times/week and ≥30 min/session).

^3^ Abdominal obesity, WC ≥ 90 cm in men and ≥85 cm in women

^4^ *P* values for interaction were obtained by including the cross-product term of the individual sex-specific non-genetic factors and sex.
