# Supplementary material for: Sex-specific associations between dietary legume subtypes and type 2 diabetes in a prospective cohort study
Source: Epidemiol Health. 2024 Oct 17;46:e2024083. doi: 10.4178/epih.e2024083 (PMC11832243; doi:10.4178/epih.e2024083)
Supplement: Supplementary Material 5. — Incidence rate ratio (IRR) and 95% confidence intervals (CI) of type 2 diabetes mellitus (T2DM) according to dietary soy consumption and genotype of sex difference in each of the three regions in MRCohort [file epih-46-e2024083-Supplementary-5.docx]

**Supplementary Material 5.** Incidence rate ratio (IRR) and 95% confidence intervals (CI) of type 2 diabetes mellitus (T2DM) according to dietary soy consumption and genotype of sex difference in each of the three regions in MRCohort

| Multivariable model^1^ | **Soy (soybeans and soy products) g/day** | | | | | | | | | | | | | |  |
| --- | --- | --- | --- | --- | --- | --- | --- | --- | --- | --- | --- | --- | --- | --- | --- |
|  | Yangpyeong | | | |  | Namwon | | | |  | Goryeong | | | | |
|  | T1 | T2 | T3 | *P* for  Interaction^2^ |  | T1 | T2 | T3 | *P* for  Interaction^2^ |  | T1 | T2 | T3 | *P* for  Interaction^2^ | |
| **rs10196939 (*LRRTM4*)** |  |  |  |  |  |  |  |  |  |  |  |  |  |  | |
| **AA, Wild-type** |  |  |  |  |  |  |  |  |  |  |  |  |  |  | |
| **MEN** |  |  |  | **0.6322** |  |  |  |  | **0.5896** |  |  |  |  | **0.9685** | |
| No. of cases / person years | 9／654 | 9／684 | 6／611 |  |  | 9／603 | 4／815 | 4／677 |  |  | 7／468 | 4／552 | 3／540 |  |  |
| Multivariable IRR^1^ | 1.00 | 0.90  (0.33-2.44) | 0.65  (0.20-2.10) |  |  | 1.00 | 0.32  (0.10-0.95) | 0.37  (0.06-2.10) |  |  | 1.00 | 0.37  (0.12-1.13) | 0.35  (0.08-1.50) |  |  |
| **WOMEN** |  |  |  |  |  |  |  |  |  |  |  |  |  |  |  |
| No. of cases / person years | 14／886 | 8／1,071 | 11／1,124 |  |  | 14／951 | 10／1,091 | 11／1,318 |  |  | 22／831 | 16／1,177 | 12／1,032 |  |  |
| Multivariable IRR^1^ | 1.00 | 0.59  (0.23-1.55) | 0.76  (0.32-1.76) |  |  | 1.00 | 0.66  (0.25-1.72) | 0.58  (0.20-1.68) |  |  | 1.00 | 0.45  (0.22-0.93) | 0.41  (0.19-0.91) |  |  |
| **AG and GG** |  |  |  |  |  |  |  |  |  |  |  |  |  |  | |
| **MEN** |  |  |  | **0.0346** |  |  |  |  | **0.0068** |  |  |  |  | **0.1488** | |
| No. of cases / person years | 8／1017 | 14／1,199 | 25／1,194 |  |  | 9／920 | 10／1,032 | 23／1,214 |  |  | 9／893 | 18／1,009 | 14／939 |  |  |
| Multivariable IRR^1^ | 1.00 | 1.43  (0.60-3.39) | 2.47  (1.08-5.63) |  |  | 1.00 | 1.00  (0.41-2.44) | 1.99  (0.82-4.80) |  |  | 1.00 | 1.75  (0.75-4.01) | 1.85  (0.71-4.48) |  |  |
| **WOMEN** |  |  |  |  |  |  |  |  |  |  |  |  |  |  |  |
| No. of cases / person years | 24／1,943 | 16／2,204 | 20／2,097 |  |  | 27／1779 | 13／2,059 | 11／1,917 |  |  | 21／1,921 | 14／1,962 | 18／2,231 |  |  |
| Multivariable IRR^1^ | 1.00 | 0.53  (0.28-1.01) | 0.55  (0.90-1.01) |  |  | 1.00 | 0.45  (0.23-0.87) | 0.39  (0.19-0.81) |  |  | 1.00 | 0.57  (0.28-1.18) | 0.66  (0.32-1.40) |  |  |
| **rs11750158 (near *GFPT2)*** |  |  |  |  |  |  |  |  |  |  |  |  |  |  | |
| **GG, Wild-type** |  |  |  |  |  |  |  |  |  |  |  |  |  |  | |
| **MEN** |  |  |  | **0.1907** |  |  |  |  | **0.2355** |  |  |  |  | **0.5258** | |
| No. of cases / person years | 6／708 | 5／874 | 9／824 |  |  | 5／653 | 7／882 | 11／892 |  |  | 3／636 | 5／677 | 3／594 |  |  |
| Multivariable IRR^1^ | 1.00 | 0.60  (0.17-2.17) | 1.11  (0.37-3.32) |  |  | 1.00 | 1.01  (0.30-3.36) | 1.60  (0.44-5.85) |  |  | 1.00 | 1.31  (0.38-4.50) | 0.88  (0.19-402) |  |  |
| **WOMEN** |  |  |  |  |  |  |  |  |  |  |  |  |  |  |  |
| No. of cases /  person years | 26／1,262 | 8／1,456 | 12／1,456 |  |  | 16／1183 | 8／1,307 | 8／1,284 |  |  | 19／1,047 | 15／1,287 | 15／1,351 |  |  |
| Multivariable IRR^1^ | 1.00 | 0.29  (0.13-0.66) | 0.35  (0.17-0.72) |  |  | 1.00 | 0.60  (0.26-1.41) | 0.58  (0.24-1.35) |  |  | 1.00 | 0.70  (0.31-1.57) | 0.76  (0.35-1.64) |  |  |
| **GA and AA** |  |  |  |  |  |  |  |  |  |  |  |  |  |  | |
| **MEN** |  |  |  | **0.8074** |  |  |  |  | **0.0667** |  |  |  |  | **0.2552** | |
| No. of cases / person years | 10／919 | 18／955 | 21／956 |  |  | 12／838 | 4／903 | 16／951 |  |  | 13／683 | 17／872 | 14／859 |  |  |
| Multivariable IRR^1^ | 1.00 | 1.76  (0.79-3.94) | 1.97  (0.87-4.47) |  |  | 1.00 | 0.33  (0.10-1.04) | 1.33  (0.57-3.09) |  |  | 1.00 | 0.96  (0.45-2.04) | 1.12  (0.48-2.61) |  |  |
| **WOMEN** |  |  |  |  |  |  |  |  |  |  |  |  |  |  |  |
| No. of cases / person years | 11／1,461 | 16／1,782 | 18／1,680 |  |  | 24／1,465 | 13／1,709 | 14／1,914 |  |  | 23／1,587 | 14／1,744 | 14／1,834 |  |  |
| Multivariable IRR^1^ | 1.00 | 1.20  (0.56-2.58) | 1.35  (0.65-2.79) |  |  | 1.00 | 0.45  (0.21-0.96) | 0.40  (0.18-0.93) |  |  | 1.00 | 0.41  (0.20-0.86) | 0.34  (0.16-0.76) |  |  |

^1^ The multivariable model was adjusted for age (years), higher education level (≥ 12 years of education), regular exercise (≥ 3 times/week and ≥ 30 minutes/session), current smoking status (yes or no), alcohol consumption (g/d), body mass index (kg/m^2^), total energy intake(kcal/d), and modified Diet Quality Index-International (DQI-I) in men and women.

^2^ *P* values for interaction were obtained by including the cross-product term of the tertile soy consumption and sex.
