# Supplementary material for: Sex-specific associations between dietary legume subtypes and type 2 diabetes in a prospective cohort study
Source: Epidemiol Health. 2024 Oct 17;46:e2024083. doi: 10.4178/epih.e2024083 (PMC11832243; doi:10.4178/epih.e2024083)
Supplement: Supplementary Material 6. — Incidence rate ratio (IRR) and 95% confidence intervals (CI) of type 2 diabetes by dietary legumes in the three cohorts [file epih-46-e2024083-Supplementary-6.docx]

**Supplementary Material 6**. Incidence rate ratio (IRR) and 95% confidence intervals (CI) of type 2 diabetes by dietary legumes in the three cohorts

| Model^1^ | Dietary legumes consumption (g/d) | | | | | | | | | |
| --- | --- | --- | --- | --- | --- | --- | --- | --- | --- | --- |
|  | MEN | | | | *P*-trend ^2^ | WOMEN | | | | *P*-trend ^2^ |
| **Soy (soybeans and soy products)** | Q1 | Q2 | Q3 | Q4 |  | Q1 | Q2 | Q3 | Q4 |  |
| **MRCohort** |  |  |  |  |  |  |  |  |  |  |
| Median intake (min-max, g/d) | 8.6 (0-15.0) | 21.3 (15.6-27.6) | 37.3 (27.6-49.8) | 75.5 (49.8-413) |  | 7.0 (0-12.1) | 17.3 (12.1-22.9) | 30.7 (22.9-43.4) | 66.6 (43.4-429) |  |
| No. of cases / person years | 45 / 4064 | 40 / 4659 | 65 / 4788 | 61 / 4647 |  | 109 / 7179 | 76 / 8005 | 68 / 8501 | 69 / 8445 |  |
| Multivariable IRR + Diet Quality Index | 1.00 | 0.74 (0.48-1.13) | 1.09 (0.73-1.65) | 1.15 (0.74-1.78) | 0.1877 | 1.00 | 0.67 (0.49-0.91) | 0.54 (0.39-0.74) | 0.52 (0.37-0.73) | 0.0022 |
| **ARIRANG** |  |  |  |  |  |  |  |  |  |  |
| Median intake (min-max, g/d) | 14.1 (0-20.6) | 27.7 (20.6-34.4) | 43.2 (24.5-55.8) | 77.1 (55.9-341) |  | 14.5 (0-21.0) | 27.1 (21.0-34.5) | 42.5 (34.5-54.9) | 81.5 (55.0-468) |  |
| No. of cases / person years | 23 / 2476 | 28 / 2905 | 31 / 2985 | 36 / 2845 |  | 33 / 4252 | 31 / 4753 | 32 / 4642 | 38 / 4584 |  |
| Multivariable IRR + Diet Quality Index | 1.00 | 0.93 (0.52-1.67) | 0.99 (0.54-1.81) | 1.14 (0.56-2.31) | 0.5724 | 1.00 | 0.74 (0.45-1.21) | 0.77 (0.47-1.26) | 0.87 (0.52-1.47) | 0.9849 |
| **Kangwha** |  |  |  |  |  |  |  |  |  |  |
| Median intake (min-max, g/d) | 8.8 (0-15.8) | 21.7 (15.8-27.5) | 35.5 (27.5-47.8) | 71.5 (47.9-519) |  | 8.5 (0-14.7) | 20.0 (14.7-25.7) | 34.0 (25.7-46.9) | 74.6 (47-1310) |  |
| No. of cases / person years | 22 / 1371 | 22 / 1639 | 18 / 1846 | 19 / 1605 |  | 26 / 2238 | 19 / 2792 | 15 / 2888 | 19 / 2839 |  |
| Multivariable IRR + Diet Quality Index | 1.00 | 0.91 (0.50-1.67) | 0.72 (0.37-1.42) | 0.85 (0.42-1.72) | 0.7020 | 1.00 | 0.56 (0.30-1.06) | 0.43 (0.22-0.84) | 0.51 (0.26-0.98) | 0.1628 |
| **Pooled-analysis**^3^ |  |  |  |  |  |  |  |  |  |  |
| Fixed effect model  Multivariable IRR + Diet Quality Index | **1.00** | **0.38 (0.61-1.11)** | **0.98 (0.72-1.33)** | **1.08 (0.78-1.49)** | **0.3931** | **1.00** | **0.67 (0.53-0.85)** | **0.57 (0.44-0.74)** | **0.59 (0.45-0.77)** | **0.0001** |
| **Beans** | T1 | T2 | T3 |  |  | T1 | T2 | T3 |  |  |
| **MRCohort** |  |  |  |  |  |  |  |  |  |  |
| Median intake (min-max, g/d) | 0 (0-0.1) | 0.4 (0.1-0.9) | 1.9 (0.9-28.1) |  |  | 0 (0-0.1) | 0.4 (0.1-0.9) | 1.6 (0.9-40.9) |  |  |
| No. of cases / person years | 70 / 5,449 | 66 / 6,280 | 75 / 6,429 |  |  | 131 / 9,402 | 106 / 11,767 | 85 / 10,959 |  |  |
| Multivariable IRR + Diet Quality Index | 1.00 | 0.76 (0.54-1.07) | 0.78 (0.55-1.11) |  | 0.3534 | 1.00 | 0.66 (0.51-0.85) | 0.58 (0.43-0.77) |  | 0.0025 |
| **ARIRANG** |  |  |  |  |  |  |  |  |  |  |
| Median intake (min-max, g/d) | 0 (0-0.2) | 0.5 (0.2-1.1) | 2.2 (1.1-49.0) |  |  | 0.1 (0-0.3) | 0.6 (0.3-1.1) | 2.3 (1.1-53.6) |  |  |
| No. of cases / person years | 43 / 3,271 | 38 / 3,995 | 37 / 3,945 |  |  | 57 / 5,345 | 42 / 6,588 | 35 / 6,298 |  |  |
| Multivariable IRR + Diet Quality Index | 1.00 | 0.67 (0.42-1.05) | 0.62 (0.38-1.01) |  | 0.1388 | 1.00 | 0.60 (0.40-0.89) | 0.47 (0.30-0.75) |  | 0.0006 |
| **Kangwha** |  |  |  |  |  |  |  |  |  |  |
| Median intake (min-max, g/d) | 0 (0-0.3) | 0.6 (0.3-1.2) | 2.3 (1.2-80.6) |  |  | 0.1 (0-0.3) | 0.7 (0.3-1.3) | 2.5 (1.3-41.0) |  |  |
| No. of cases / person years | 35 / 1,831 | 23 / 2,269 | 23 / 2,361 |  |  | 32 / 3,007 | 22 / 3,913 | 25 / 3,835 |  |  |
| Multivariable IRR + Diet Quality Index | 1.00 | 0.55 (0.32-0.93) | 0.51 (0.29-0.89) |  | 0.0505 | 1.00 | 0.52 (0.30-0.91) | 0.55 (0.31-0.95) |  | 0.1009 |
| **Pooled-analysis**^3^ |  |  |  |  |  |  |  |  |  |  |
| Fixed effect model  Multivariable IRR + Diet Quality Index | **1.00** | **0.68 (0.54-0.87)** | **0.67 (0.52-0.87)** |  | **0.0064** | **1.00** | **0.62 (0.51-0.76)** | **0.54 (0.43-0.68)** |  | **<.0001** |
| **Peanuts** | T1 | T2 | T3 |  |  | T1 | T2 | T3 |  |  |
| **MRCohort** |  |  |  |  |  |  |  |  |  |  |
| Median intake (min-max, g/d) | 0 (0-0) | 0.2 (0.1-0.3) | 1 (0.4-20.7) |  |  | 0 (0-0) | 0.1 (0.1-0.2) | 0.8 (0.2-13.5) |  |  |
| No. of cases / person years | 87 / 6840 | 45 / 4929 | 79 / 6389 |  |  | 183 / 15141 | 43 / 5454 | 96 / 11533 |  |  |
| Multivariable IRR + Diet Quality Index | 1.00 | 0.68 (0.48-0.97) | 0.87 (0.63-1.20) |  | 0.7921 | 1.00 | 0.71 (0.51-0.98) | 0.76 (0.57-1.00) |  | 0.1069 |
| **ARIRANG** |  |  |  |  |  |  |  |  |  |  |
| Median intake (min-max, g/d) | 0 (0-0) | 0.2 (0.1-0.3) | 0.9 (0.4-33.8) |  |  | 0 (0-0) | 0.2 (0.1-0.4) | 1.2 (0.4-33.8) |  |  |
| No. of cases / person years | 55 / 4,176 | 20 / 3,174 | 43 / 3,861 |  |  | 66 / 6,655 | 28 / 4,912 | 40 / 6,663 |  |  |
| Multivariable IRR + Diet Quality Index | 1.00 | 0.48 (0.29-0.81) | 0.85 (0.53-1.34) |  | 0.9282 | 1.00 | 0.58 (0.37-0.90) | 0.63 (0.40-1.00) |  | 0.1656 |
| **Kangwha** |  |  |  |  |  |  |  |  |  |  |
| Median intake (min-max, g/d) | 0 (0-0) | 0.2 (0.1-0.2) | 0.8 (0.2-22.5) |  |  | 0 (0-0) | 0.1 (0.1-0.2) | 0.9 (0.2-19.7) |  |  |
| No. of cases / person years | 50 / 2,713 | 5 / 1,246 | 26 / 2,502 |  |  | 44 / 4,569 | 9 / 2,206 | 26 / 3,980 |  |  |
| Multivariable IRR + Diet Quality Index | 1.00 | 0.22 (0.90-0.55) | 0.62 (0.37-1.02) |  | 0.2122 | 1.00 | 0.44 (0.22-0.91) | 0.77 (0.47-1.29) |  | 0.6466 |
| **Pooled-analysis**^3^ |  |  |  |  |  |  |  |  |  |  |
| Fixed effect model  Multivariable IRR + Diet Quality Index | **1.00** | **0.48 (0.28-0.81)** | **0.80 (0.63-1.01)** |  | **0.2824** | **1.00** | **0.63 (0.49-0.80)** | **0.73 (0.59-0.90)** |  | **0.0181** |

^1^ Multivariable model was adjusted for age (years), higher education level (≥ 12 years of education), regular exercise (≥ 3 times/week and ≥ 30 minutes/session), smoking status (never/former/current), alcohol consumption (g/d), Body Mass Index (kg/m^2^), total energy intake(kcal/d), and modified Diet Quality Index-International (DQI-I) score in men and women.

^2^ *P* values for linear trend were obtained by imputing the median value of each quartile (or tertile) and treating it as a continuous variable using a modified Poisson regression with a robust error estimator.

^3^ The pooled IRRs form the multivariable-adjusted models across the 3 cohorts were combined using an inverse variance-weighted fixed-effects mata-analysis
